# An adaptive method of defining negative mutation status for multi-sample comparison using next-generation sequencing

**DOI:** 10.1186/s12920-021-00880-8

**Published:** 2021-12-02

**Authors:** Nicholas Hutson, Fenglin Zhan, James Graham, Mitsuko Murakami, Han Zhang, Sujana Ganaparti, Qiang Hu, Li Yan, Changxing Ma, Song Liu, Jun Xie, Lei Wei

**Affiliations:** 1 Department of Biostatistics and Bioinformatics, Roswell Park Comprehensive Cancer Center, Buffalo, NY USA; 2 PET/CT Center, The First Affiliated Hospital of USTC, Division of Life Sciences and Medicine, University of Science and Technology, Hefei, 230001 China; 3 Center for Personalized Medicine, Roswell Park Comprehensive Cancer Center, Buffalo, NY USA; 4 Department of Chemistry and Physics, Indiana State University, Terre Haute, IN USA; 5 Department of Biostatistics, University At Buffalo, Buffalo, NY USA; 6 Department of Statistics, Purdue University, West Lafayette, IN USA

**Keywords:** Negative status, Tumor heterogeneity, Liquid biopsy, Next-generation sequencing, Genetic testing, Personalized medicine

## Abstract

**Background:**

Multi-sample comparison is commonly used in cancer genomics studies. By using next-generation sequencing (NGS), a mutation's status in a specific sample can be measured by the number of reads supporting mutant or wildtype alleles. When no mutant reads are detected, it could represent either a true negative mutation status or a false negative due to an insufficient number of reads, so-called "coverage". To minimize the chance of false-negative, we should consider the mutation status as "unknown" instead of "negative" when the coverage is inadequately low. There is no established method for determining the coverage threshold between negative and unknown statuses. A common solution is to apply a universal minimum coverage (UMC). However, this method relies on an arbitrarily chosen threshold, and it does not take into account the mutations' relative abundances, which can vary dramatically by the type of mutations. The result could be misclassification between negative and unknown statuses.

**Methods:**

We propose an adaptive mutation-specific negative (MSN) method to improve the discrimination between negative and unknown mutation statuses. For a specific mutation, a non-positive sample is compared with every known positive sample to test the null hypothesis that they may contain the same frequency of mutant reads. The non-positive sample can only be claimed as “negative” when this null hypothesis is rejected with all known positive samples; otherwise, the status would be “unknown”.

**Results:**

We first compared the performance of MSN and UMC methods in a simulated dataset containing varying tumor cell fractions. Only the MSN methods appropriately assigned negative statuses for samples with both high- and low-tumor cell fractions. When evaluated on a real dual-platform single-cell sequencing dataset, the MSN method not only provided more accurate assessments of negative statuses but also yielded three times more available data after excluding the “unknown” statuses, compared with the UMC method.

**Conclusions:**

We developed a new adaptive method for distinguishing unknown from negative statuses in multi-sample comparison NGS data. The method can provide more accurate negative statuses than the conventional UMC method and generate a remarkably higher amount of available data by reducing unnecessary “unknown” calls.

## Background

Multi-sample comparison is commonly used in cancer genomics analyses, such as tumor heterogeneity [1–3] and serial liquid biopsies [4–6]. An essential part of these multi-sample analyses is to compare the mutational profiles between multiple samples from the same patient [2]. As a consequence of the genetic evolution or environmental factors, a mutation from one sample could either be positive or negative in another sample of the same patient. Accurate assessment of mutation statuses in all related samples not only provides essential information for deconvoluting tumor progression, but also plays an important role in identifying treatment targets [2, 7, 8]. However, similar to any other tests, a genetic test may generate a non-informative result due to technical failure. These non-informative results can be misclassified as negative and lead to incorrect mutation profiles, which may compromise the downstream effort for developing personalized treatment [9]. To accurately assess negative status, a designated strategy is needed to identify the potential false negative calls and reclassify them to "unknown" status.

Next-generation sequencing (NGS) technology has made it feasible to characterize somatic mutations in large quantities efficiently [10, 11]. A common technical failure for identifying a mutation using NGS is low coverage, which refers to the number of NGS reads covering the genomic site of the mutation. When a mutation is not detected, determining whether the coverage is sufficient for claiming a negative status becomes a major challenge. A common bioinformatics method to avoid false-negative is to require a universal minimum coverage (UMC) [12]. Other alternative methods have also been used, such as providing an overall justification of low false-negative rate by computational simulation [13] or adopting ultradeep sequencing to reduce false-negative [14]. Theoretically, the minimum coverage needed to claim a negative status of a specific mutation should depend on the relative abundance of that mutation. In high-purity tumors, the mutations are easier to detect and thus require less coverage to claim negative status. On the other hand, for the samples with low levels of tumor content such as circulating cell-free DNA (cfDNA), much higher coverage is needed to claim negative status [15]. However, none of the existing methods take account of the difference in the relative abundance of individual mutations when determining the minimum coverage threshold. In NGS, a mutation's relative abundance can be measured by the Variant Allele Frequency (VAF), defined as the fraction of NGS reads harboring the mutant allele at the mutation's site.

In a hypothetical example for demonstration purposes, a non-small cell lung cancer patient received the first biopsy test, which identified two actionable *EGFR* mutations [16]: T790M at 56% VAF and a C797S at 5% VAF. After treatment, a follow-up biopsy did not detect any of these two mutations. However, the coverage was suboptimal in the second biopsy (only 15X and 30X for T790M and C797S, respectively) (Table 1). When using the UMC method with a threshold of 20X, the C797S mutation would be classified as negative, while the T790M mutation would be classified as unknown (Status #1). A problem with this strategy of solely defining negative status using UMC is that the mutation's relative abundance, in the form of VAF, was not taken into consideration. In the first biopsy, the T790M mutation had a higher VAF and thus required less coverage to detect. However, there is no perfect way of determining the expected VAF in the second biopsy. To solve this problem by developing a more adaptive method than UMC, we propose an alternative approach. Instead of asking whether the second biopsy is negative, which cannot be answered definitively, we now ask whether the second biopsy appears to be similar to the first biopsy in terms of the mutation's relative abundance. When this null-hypothesis is rejected, it suggests the second biopsy is different from the first positive biopsy. We then define the second biopsy as "negative", given the fact that no mutant reads have been detected in it, and there are sufficient non-mutant reads to show it is significantly different from the known positive biopsy. With this strategy, the T790M mutation was determined to be negative (p < 0.05, Fisher’s exact test), while the C797S was classified as unknown (p > 0.05) (Status #2). Without considering the mutation’s relative abundance, the conventional UMC method tends to unnecessarily misclassify low-VAF mutations as negative; meanwhile, it can be over-conservative for expected high-VAF mutations by failing to call them as negative. In this example, the MSN method identified the *EGFR* C797S as “unknown” in the second biopsy because of a previous low-VAF positive result, suggesting additional coverage would be needed to determine its actual status in the second biopsy. Further, it rescued the high-VAF mutation (T790) from the non-informative “unknown” status and reclassified it as “negative”. These assessments generated by the MSN method were more seasonable as they matched the specific characteristics of the mutations.

**Table 1:** A hypothetical example of the negative data problem

Mutations	Biopsy (#1)			Biopsy (#2)			
	Read counts (mutant/total)	VAF (%)	Status	Read counts (mutant/total)	VAF (%)	Status #1 (UMC 20X)	Status #2 (MSN)
*EGFR* (T790M)	40/72	56	Positive	0/15	0	Unknown	Negative
*EGFR* (C797S)	5/100	5	Positive	0/30	0	Negative	Unknown

## Methods

### Introduction

We propose a designated method for distinguishing "unknown" from "negative" status by individual mutations, which incorporates information from the observed mutation-positive (referred to as "positive") samples. In general, a mutation can be classified as "positive" when there is solid evidence that mutant reads are present. On the other hand, when no mutant reads are detected, the absence of mutant reads could either be true negative or false negative due to insufficient numbers of measures (i.e., low coverage in NGS data). Our goal is to identify those samples at high risk of being false negative and reclassify them as "unknown". The MSN method allows the users to use any preferred method for defining positive statuses, such as an existing mutation caller or any customized threshold such as a minimum number of mutant reads or a minimum mutant VAF. After positive samples are defined, the focus of the MSN method is in distinguishing "unknown" from "negative" statuses for the remaining "non-positive" samples.

The proposed method is based on a central hypothesis that a potential false negative sample may contain the same frequency of mutant reads as one of the positive samples but was not tested positive because of insufficient numbers of measures, i.e., low coverage. The common observation of mutations present in different samples at the same or approximate VAFs [2] provides an opportunity for developing a mutation-specific strategy for determining the range of expected relative abundance. In the proposed MSN method, any "non-positive" sample will be tested against every positive sample for the null-hypothesis that the "non-positive" sample may contain the same VAF of mutant reads as the "positive" sample. If this null-hypothesis is rejected when comparing the “non-positive” sample with all known positive samples, then the mutation status of the non-positive sample is defined as "negative". Otherwise, it is considered as "unknown". In test runs using both simulated and a real dataset of dual-platform single-cell sequencing data, our method demonstrated improved performance compared with existing methods using UMC.

### Data preparation

The current method was designed specifically for any dataset containing multiple tumor samples from the same patient, referred to as "related" samples. The applicable samples may also include other samples containing tumor cells or DNA, such as circulating tumor cells (CTCs) and circulating tumor DNA (ctDNA). Before running the current method, we assume the following steps have already been finished: (1) identify somatic mutations from all tumor samples using any variant caller chosen by the user; (2) combine the mutations from all "related" samples of the same patient into a list of unique mutations, as defined by the chromosome, position, reference allele, and mutant allele; 3) calculate the numbers of mutant and wildtype reads of each unique mutation in all "related" samples using any appropriate program such as the SAMtools [17], and define which samples are positive for each mutation. The input data to our pipeline is a data matrix containing the counts of mutant and wildtype reads of every mutation in all "related" samples and which samples are considered as positive.

### Performance evaluation using simulated data

Tumor BAM files containing varying fractions of tumor cells were simulated using BAMSurgeon [18]. High-quality paired-end reads passing Illumina RTA filter were initially processed against the NCBI human reference genome (GRCh37) using public available bioinformatics tools [19, 20], and Picard (http://picard.sourceforge.net/). A total of 200 somatic mutations from a previously published study [21] were introduced to an unrelated wildtype BAM [22]. To consider tumor heterogeneity, we split the 200 somatic mutations into two groups: clonal mutations (n = 100) that are present in all samples and subclonal mutations (n = 100) that are randomly present in half of the samples. An overall 200X coverage was used for all BAM files. We assumed all mutations are located in diploid regions, i.e., the mutation’s expected VAF is half of the tumor cell fraction in the sample. For any specific mutation, the numbers of mutant reads were first calculated by multiplying the coverage with the expected VAF, with the random variation modeled using a Poisson distribution.

We simulated four different scenarios (#1–4) containing different tumor cell fractions from 90, 20, 5 to 1%. Each scenario was independently simulated three times (referred to as measurements) to mimic multiple sampling. After the simulation, the numbers of mutant and wildtype reads of each mutation in every measurement were extracted using a customized Perl program. For any measurement, any mutations with mutant reads detected are considered as mutation-positive. The remaining mutations are considered as “non-positive” and subsequently being further classified into negative or unknown statuses using different negative-defining methods. Mutations without any mutant reads in all three measurements were excluded from the downstream evaluation of negative-defining methods, as these mutations would not have been captured by any somatic mutation caller.

### Performance evaluation using real data

For the current evaluation, we used an input dataset generated by a previous study [22] for evaluating dual-platform single-cell whole-exome sequencing (Additional file 1: Table S1). After single-cell capture and whole-genome amplification (WGA), each cell was captured using two platforms, including *Agilent* SureSelect XT Target Enrichment System (referred to as "AGL") and *Illumina* Nextera rapid capture (referred as "NXT"), to create two independent samples from every cell. Somatic mutations were identified from individual samples. Subsequently, all identified somatic mutations were consolidated into a list of unique mutations. Then every unique mutation was re-examined in all samples to extract the counts of mutant and wildtype reads. In an ideal error-free experiment, the two paired runs (AGL and NXT) of the same cell should yield identical mutation status for any specific mutation. Based on this principle, this dataset is used as the gold standard to evaluate the performance of the two negative-defining methods (UMC and MSN) by (1) the total number of informative data points, defined as the single-cell/mutation pairs where both platforms (NXT and AGL) yielded an informative mutation status after excluding “unknown” statuses; (2) the concordance between the two platforms (NXT and AGL), defined as the percentage of informative data points where the two platforms yielded the same mutation statuses in one single cell. Here, all unique mutations that were initially identified from at least one cell were included, then re-visited in all available cells to extract the numbers of mutant and wildtype reads for subsequent mutation-status analysis.

## Results

### Demonstrate the process for defining mutation status in the MSN method

**Fig. 1:**
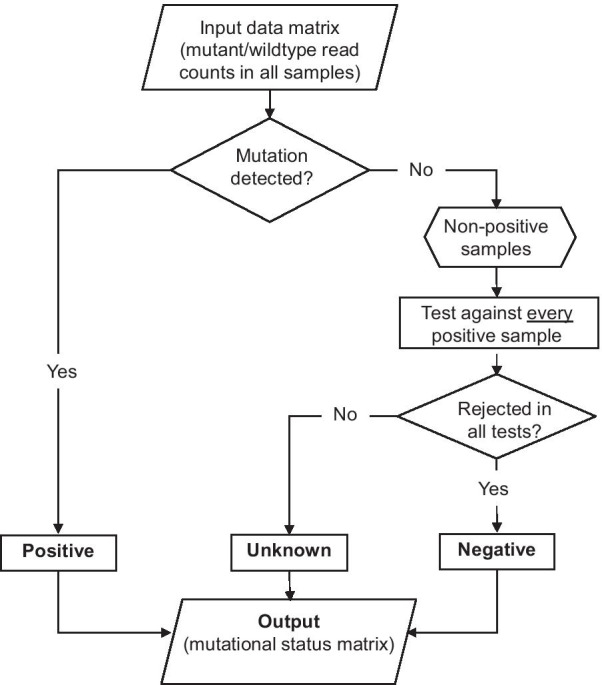
Flowchart illustrating the process of defining a mutation’s status in multiple related samples. Begin with an input data matrix containing the numbers of mutant and wildtype read counts for every mutation in all related samples (top), the mutation's status in each sample is classified in a two-step fashion. First, positive sample/mutation pairs were identified. We assume the users have completed this step using their preferred method before running MSN. The MSN method does not create or remove positive statuses but directly report them to the output (left). Second, for every mutation, each “non-positive” sample is compared with every positive sample to determine if they may contain the same frequency of mutant reads. If and only if this null hypothesis is rejected against all positive samples, then this non-positive sample is considered as negative (right), otherwise, it would be classified as unknown due to low coverage (middle). The output is a data matrix containing all updated mutation statuses (bottom)

The process for defining mutation status using the proposed adaptive MSN is illustrated in Fig. 1: (1) Defining "positive" samples. For any mutation, before defining negative samples, we need first to identify the "positive" samples. There are numerous published tools available for identifying mutation-positive samples [23–27]. To use the MSN method, we assume the users have already identified the positive samples using their own preferred method. (2) Classify "non-positive" samples into "negative" and "unknown" groups. The remaining "non-positive" samples are further classified into two groups, "negative" and "unknown" using the MSN method. Specifically, each "non-positive" sample was tested against every "positive" sample to exclude the possibility that the absence of mutant reads was a false negative due to low coverage. The null hypothesis is that the "non-positive" sample may contain an equal proportion of mutant reads as a given positive sample. If the null-hypothesis is successfully rejected in tests against all positive samples, then that "non-positive" sample is defined as "negative". Otherwise, if we fail to reject the null-hypothesis against any one of the positive samples, then that non-positive sample is considered as "unknown". For "unknown" samples, additional coverage is required to determine its true status. In this evaluation, the null hypothesis was tested using Fisher's exact test on the read count data; specifically, the numbers of mutant and wildtype reads in the two samples to be compared.

This negative-defining process using the MSN method is demonstrated in a hypothetical example (Table 2). A mutation was examined in five separate samples (A-E) with the numbers of mutant and wildtype reads listed in all samples. Following the above criteria, two of five samples (A and C) were identified as "positive" (Table 2A). The remaining three "non-positive" samples (B, D, and E, marked as "TBD" for "to-be-determined") then went through the following process to distinguish "negative" from "unknown" samples. To test the null hypothesis that a "non-positive" sample may contain an equal frequency of mutant reads as any of the known "positive" samples, we compared every "non-positive" sample (B, D, or E) with each of the two "positive" samples (A and C) using Fisher's exact test. At least one test turned out to be not significant for sample D (D vs. C) and E (E vs. A, and E vs. C). In such cases, we could not exclude the possibility that samples D and E may contain the same frequency of mutant reads as at least one of the "positive" samples. Therefore, samples D and E were classified as "unknown". For the remaining sample B, since a significant difference (p < 0.05) was found, and the null-hypothesis was rejected against all positive samples, sample B was classified as "negative" (Table 2B). Adjustment of the p-value cutoff for multiple testing is not considered here. We will allow users to vary the p-value cutoff in practice.

**Table 2:** A step-by-step example of differentiating “unknown” from “negative” status using the MSN method

*A. Define* “*positive*” *samples*			
Sample	Read counts		Status
	Mutant	Total (coverage)	
A	10	20	Positive
B	0	20	TBD*
C	4	9	Positive
D	0	8	TBD*
E	0	5	TBD*

*B. Separate* “*unknown*” *from* “*negative*” *statuses*			
“Non-positive” sample	Rejected against positive samples (p value**)		Final status
	vs A	vs C	
B	Yes (< 0.01)	Yes (< 0.01)	Negative
D	Yes (< 0.05)	No	Unknown
E	No	No	Unknown

^*^TBD to-be-determined

^**^By Fisher's exact test

### Evaluate negative-defining methods using simulated datasets

We evaluated the performance of MSN in simulated datasets. To determine the effect of the relative abundance of mutations on the performance of MSN, we simulated four different scenarios containing varying tumor cell fractions from 90, 20, 5 to 1%. This wide fraction range allows us to mimic many real situations of mutation detection including clonal mutations in high-quality tumor biopsy (90%), subclonal mutations or mutations within CNV regions (20%), and low-frequency mutations in rare tumor population or liquid biopsy (5% and 1%). At each tumor cell fraction, three datasets were independently simulated (referred to as “measurements”) to mimic multiple samplings such as multisite biopsy or longitudinal liquid biopsies. In each measurement, 200 pre-selected mutations, including 100 clonal mutations and 100 subclonal mutations, were introduced into a wildtype BAM file. After all positive mutations were identified in individual measurements, we discriminated between the negative and unknown statuses using either MSN or UMC methods with different cutoffs, including two MSN cutoffs (p < 0.01 and p < 0.05) and four UMC cutoffs (20X, 50X, 200X and 300X).

**Fig. 2:**
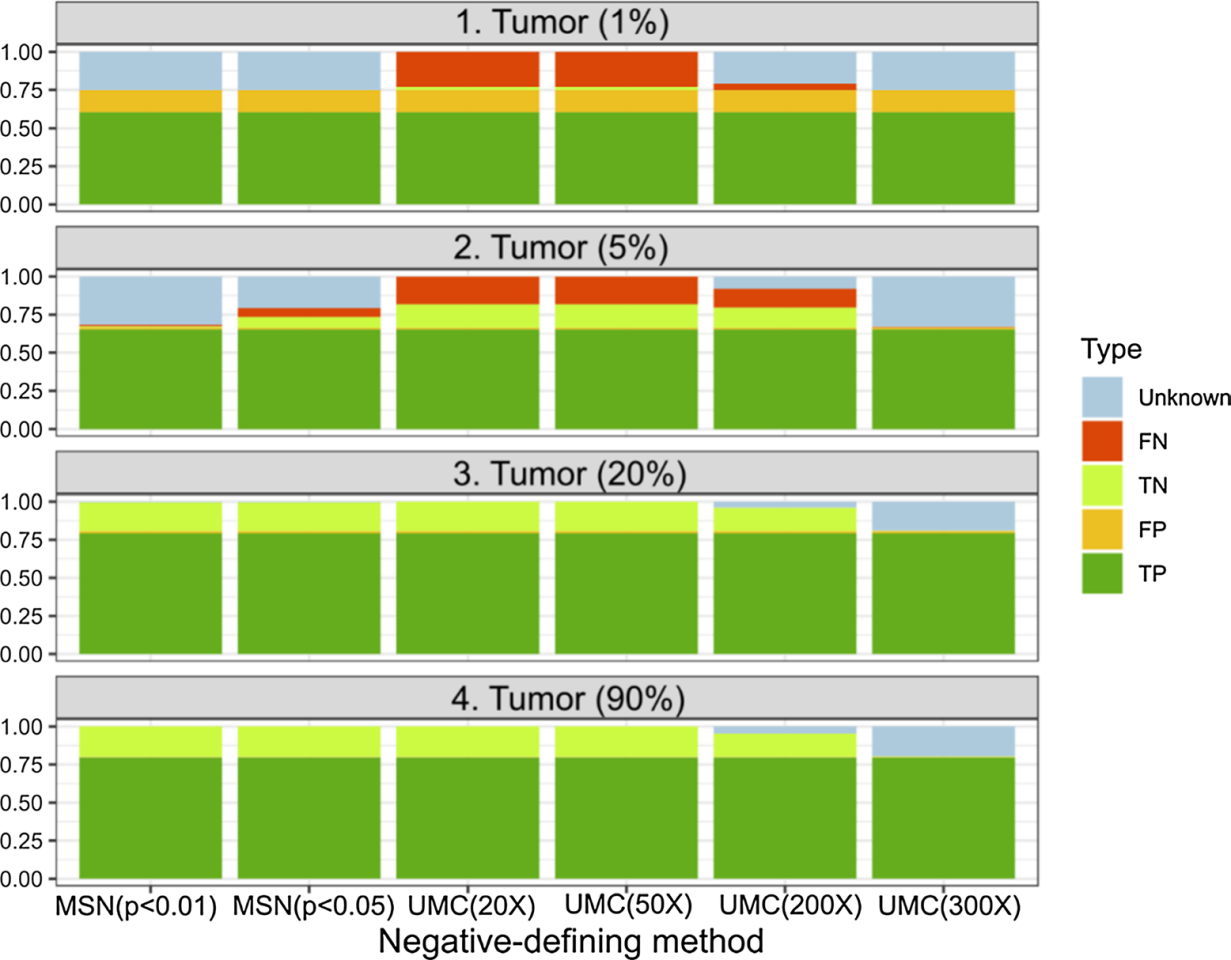
Evaluate the performance of negative-defining methods in a simulated dataset. From bottom to top: we simulated four different scenarios containing varying tumor cell fractions from 90, 20, 5 to 1%. Each scenario was independently simulated three times (referred to as measurements) to mimic multiple sampling. Only mutations that are positive in at least one of the three measurements after simulation were included. X-axis: different negative-defining methods including MSN using two thresholds (p < 0.01 and p < 0.05) and UMC using four thresholds (minimum coverage for non-positive samples to be considered as negative: 20X, 50X, 200X and 300X). Y-axis: percent of defined mutation statuses by type (Unknown: non-positive but the coverage was too low to be considered as negative; *FN* false negative, *TN* true negative, *FP* false positive, *TP* true positive). Please note that the current negative-defining methods do not affect positive mutation statuses (TP and FP)

A clear association between tumor cell fraction and the performance of the negative-defining methods was observed (Fig. 2). When tumor cell fractions were relatively high (≥ 20%), the high-cutoff UMC methods appeared to be over-conservative. For example, in scenario #4 of 90% tumor cell fraction, the UMC 300X method incorrectly assigned 19.6% of all mutations as “unknown”, which all should be negative. On the other hand, the low-cutoff UMC methods (20X and 50X) and MSN methods correctly assigned almost all mutation statuses. In contrast, for the scenarios of very low tumor cell fractions (≤ 5%), however, the low-cutoff UMC methods (20X and 50X) resulted in dramatically increased false-negative calls. For instance, in scenario #1 of 1% tumor cell fraction, the UMC 20X and 50X assigned 25% of all mutations as negative, while most of them (22.9% of 25%) were false negative.

In contrast, the MSN methods (p < 0.01 and p < 0.05) and the UMC 300X method correctly assigned these non-positive mutations as “unknown”, which raises the concern that additional sequencing is needed to determine the mutations’ actual statuses. Overall, only the MSN methods can appropriately assign negative statuses for both high- and low- tumor cell fractions. Lastly, since the MSN method was designed for multi-sample (≥ 2) comparison, it would be of interest to determine the minimum number of measurements needed for MSN. We performed another simulation using only two measurements per scenario, and the results were highly similar to the 3-measurement comparison (Additional file 2: Figure S1). Together, these results indicate that MSN is an adaptive method that can work seamlessly with mutations of different relative abundances, which cannot be appropriately handled by the UMC method.

### Evaluation using real single-cell dual-platform sequencing data

We compared the performance of the MSN method with the conventional UMC method on an existing dataset of somatic mutations identified from the whole-exome sequencing of single cells (n = 12) derived from spheres grown from a melanoma specimen [22]. Every single cell was sequenced using two different capture methods, Illumina Nextera rapid capture ("NXT") and Agilent SureSelect XT Target Enrichment System ("AGL"). The data generated from a specific cell captured by one platform was treated as a sample, totaling 24 samples with two samples from each cell. This single-cell dual-platform dataset provided a unique opportunity for evaluating the performance of methods for defining mutation status—since the same cell was sequenced with two separate platforms, every mutation's statuses in the two samples of the same cell should match with each other, and therefore the dataset serves as a gold standard. Only the comparisons between two samples of the same cell but not cross-cell comparisons were included in the gold standard due to the cell-to-cell heterogeneity.

From these 12 cells, we identified a total of 3511 unique mutations. For each mutation, the numbers of mutant and wildtype reads were re-measured in all 24 samples (Additional file 1: Table S1). We first identified "positive" samples by requiring a minimum of two mutant reads. Subsequently, for other non-positive samples, we applied the MSN method, as well as the conventional UMC method, to distinguish "unknown" from "negative" statuses. For ease of communication, the final mutation status of one specific mutation in a given cell will be henceforth regarded as a “data point”. After excluding all "unknown" statuses due to low coverage in either platform, the performance of the two negative-defining methods was assessed by (1) the concordance between the two capture platforms of AGL and NXT, defined as the percentage of “data points” where the two paired runs, i.e., NXT and AGL of the same cell, generated the same status (either "positive" or "negative") for the same mutation; (2) the total numbers of “informative data points”, where an informative data point was defined as any”data point” with an either “positive” or “negative” status (excluding "unknown"). In general, a more conservative negative-defining method would classify more "negative" statuses as "unknown", leading to a higher concordance but at the cost of a reduced number of “informative data points”.

**Fig. 3:**
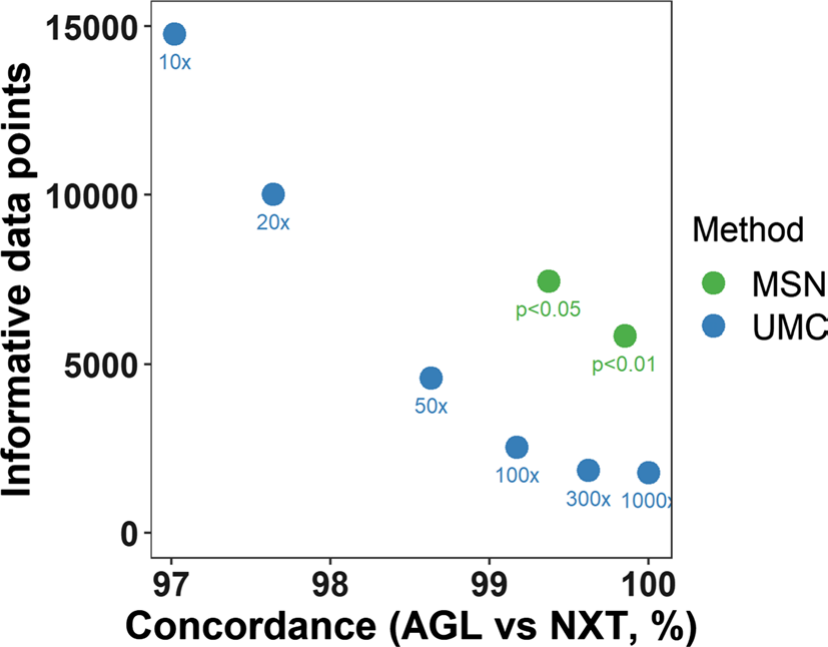
Evaluate the performance of negative-defining methods using dual-platform single-cell sequencing data. Two negative-defining methods, UMC and MSN, were tested in a single-cell dual-platform whole-exome sequencing dataset using varying thresholds, including UMC (p < 0.01, p < 0.05) and MSN (minimum coverage of 10X, 20X, 50X, 100X, 300X and 100X), as indicated under each dot. The overall performance of each method with a specific threshold was evaluated by (1) Y-axis: the total number of informative data points after excluding unknown statuses, with “informative data points” defined as the single-cell/mutation pairs where both platforms (NXT and AGL) yielded an informative mutation status, i.e., either positive or negative, but not unknown; (2) X-axis: the concordance of mutation statuses between the two platforms (NXT and AGL), defined as the percentage of informative data points where the two platforms yielded the same mutation status, either both positive or both negative, for the same single cell

In the current evaluation, we tested the two methods with the following thresholds: 1) the UMC methods with six coverage thresholds: 10X, 20X, 50X, 100X, 300X and 1000X; 2) the adaptive MSN method with two thresholds: p < 0.05 and p < 0.01(Fig. 3). For the UMC methods, as the coverage threshold increases from 10 to 1000X, the concordance increases from 97.02% to 100.00%, while the total “informative data points” decreases from 14,777 to 1790. For the MSN method, the concordances were 99.37% and 99.85%, and the total “informative data points” were 7449 and 5832, for p < 0.05 and p < 0.01, respectively. When compared at similar levels of concordance, the MSN method yielded much higher numbers of “informative data points” than the UMC method: for example, the UMC method at 100X threshold generated a concordance of 99.17% with 2543 “informative data points”. Meanwhile, the MSN method with p < 0.05 generated even better concordance (99.37%), and yielded 2.9 times more (n = 7449) “informative data points”. At a higher coverage threshold of 300X, the UMC method had increased concordance (99.62%) but dramatically reduced “informative data points” (n = 1850). For comparison, the MSN method with p < 0.01 yielded better concordance (99.85%) and produced 3.2 times more “informative data points” (n = 5832) (Additional file 1: Table S2). These results suggest that the MSN method not only provides a more accurate assessment of the negative status but also recovered remarkably higher numbers of available data (approximately three times in the current test data).

## Discussion

Multi-sample comparison on the mutational level plays an important role in understanding tumor evolution and progression [1, 2, 28]. The presence or absence of a particular mutation may affect a patient's clinical classification, such as drug resistance and metastatic potentials [16]. Therefore, accurate determination of mutation statuses is essential for any genetic testing. Although a large number of methods have been developed to identify positive samples [23–27, 29], to the best of our knowledge, there has been no existing method designed for defining negative samples or discriminating between negative status and unknown status. Most previous tumor heterogeneity studies simply required a universal minimum coverage threshold [12]. Applying a higher requirement of coverage helps reduce false-negative but result in less available data points. Theoretically, there is no universal minimum coverage for claiming the negative status as it depends on the mutation's relative abundance, which varies greatly and can be measured by VAF using NGS data.

We developed the current approach to discriminate between "unknown" and "negative" statuses based on the relative abundance information in the "positive" samples. Unlike “positive” mutation status, which only requires the presence of mutant reads to claim, a negative status cannot be definitively determined unless hypothetically every cell in the sample has been tested. We propose to define an adaptive “negative” status when a sample has no mutant reads detected and is significantly different from all known positive samples. Based on the evaluation using both simulated and real single-cell dual-platform sequencing datasets, the MSN method outperformed the conventional UMC method by providing more accurate negative statuses, meanwhile yielded more available data. The MSN method’s ability to adjust for varying VAFs is especially important for tumor analyses. Due to intratumoral heterogeneity and copy number variants (CNVs), tumor samples often contain mutations at very different levels of VAFs. In such cases, all mutations’ negative statuses cannot be correctly defined using a universal coverage cutoff. Instead, the MSN method would provide a more adaptive and accurate assessment of negative statuses. The current MSN method would be particularly useful for comparing the mutational profiles of two or more tumor samples from the same patient, serial liquid biopsies, or comparing exome and RNASeq of the same sample.

The current method has several limitations. First, MSN focuses on the discrimination between negative and unknown statuses but does not create or remove positive statuses. Instead, it relies on the user-provided positive statuses. If the user-provided input data contain false-positive for certain mutation, we expect MSN to become over-conservative and more likely to classify that mutation as unknown instead of negative in other samples. Next, our method is built on the assumption that the mutation' relative abundance is overall consistent in the tested samples. While this generally holds for the majority of mutations in cancer, the performance may be suboptimal in certain cancers containing genomic regions with excessive ongoing changes of copy number variations (CNVs) or inter-tumoral heterogeneity. Even for these challenging situations, we expect the MSN method may still perform better than the UMC method, which does not consider the variation of VAFs. Further, our method is based on the read count data generated by next-generation sequencing; therefore, it would not work for Sanger sequencing data. Lastly, the MSN method was designed for any multi-sample comparison and can work with as few as two samples (Additional file 2: Figure S1). However, the UMC method might be the only available option for single-sample analysis.

## Conclusions

We present the first method designated for reclassifying potential false negative mutation status caused by low coverage into "unknown" status in multi-sample comparison analyses. Instead of using a universal minimum coverage, our method is designated to be flexible towards individual mutations. The method has been shown to provide a more accurate assessment of negative statuses as it is adaptive to varying VAFs and yield a remarkably higher amount of available data by reducing unnecessary “unknown” calls.

## Supplementary Information

**Additional file 1. **Supplementary tables. **Table S1:** The list of somatic mutations previously identified from dual-platform single-cell whole-exome sequencing used for the current evaluation. **Table S2:** The summary of the comparison between the Agilent- and Nextera-runs using different negative-defining methods.

**Additional file 2.** Supplementary figures. **Figure S1:** Two-measurement simulation to evaluate the performance of negative-defining methods.

## Data Availability

All data generated or analyzed during this study are included in this published article and its supplementary information files. Picard was downloaded from: http://picard.sourceforge.net/. A Perl implementation of the MSN algorithm is available at https://github.com/leiwei-bioinfo/Negative_Data.

## Abbreviations

UMC: Universal minimum coverage
MSN: Mutation-specific negative
NGS: Next-generation sequencing
cfDNA: Circulating cell-free DNA
VAF: Variant allele frequency
WGA: Whole-genome amplification
NXT: Nextera rapid capture
AGL: Agilent SureSelect XT Target Enrichment System
CNVs: Copy number variations
