# Supplementary material for: An adaptive method of defining negative mutation status for multi-sample comparison using next-generation sequencing
Source: BMC Med Genomics. 2021 Dec 2;14(Suppl 2):32. doi: 10.1186/s12920-021-00880-8 (PMC8638096; doi:10.1186/s12920-021-00880-8)

Supplementary information for

An adaptive method of defining negative mutation status for multi-sample comparison using next-generation sequencing

Nicholas Hutson[1,*], Fenglin Zhan[1,2,*], James Graham[1], Mitsuko Murakami[3,4], Han Zhang[5], Sujana Ganaparti[1], Qiang Hu[1], Li Yan[1], Changxing Ma[5], Song Liu[1], Jun Xie[6,§], Lei Wei[1,§]

[1]Department of Biostatistics and Bioinformatics, [3]Center for Personalized Medicine, Roswell Park Comprehensive Cancer Center, Buffalo, NY.

[2]PET/CT center, The First Affiliated Hospital of USTC, Division of Life Sciences and Medicine, University of Science and Technology of, Hefei, China, 230001

[4]Department of Chemistry and Physics, Indiana State University, Terre Haute, IN

[5]Department of Biostatistics, University at Buffalo, Buffalo, NY.

[6]Department of Statistics, Purdue University, West Lafayette, IN

[*] N.H. and F.Z. contributed equally to this work

[§] Correspondence authors

Email addresses:

NH: ndhutso@gmail.com

FZ: zhan209@hotmail.com

JG: james.graham@stonybrookmedicine.edu

MM: mitsuko.korobkin@indstate.edu

HZ: hzhang39@buffalo.edu

SG: ganaparti.sujana@roswellpark.org

QH: qiang.hu@roswellpark.org

LY: li.yan@roswellpark.org

CM: cxma@buffalo.edu

SL: song.liu@roswellpark.org

JX: junxie@purdue.edu

LW: lei.wei@roswellpark.org

Figure Legends

Figure S1. Two-measurement simulation to evaluate the performance of negative-defining methods.

The current simulation was generated to determine if the MSN method works with as few as two "related" samples. From bottom to top: we simulated four different scenarios containing varying tumor cell fractions from 90%, 20%, 5% to 1%. Each scenario was independently simulated two times (referred to as measurements) to mimic multiple sampling. Only mutations that are positive in at least one of the three measurements after simulation were included. X-axis: different negative-defining methods including MSN using two thresholds ($p<0.01$ and $p<0.05$) and UMC using four thresholds (minimum coverage for non-positive samples to be considered as negative: 20X, 50X, 200X and 300X). Y-axis: percent of defined mutation statuses by type (Unknown: non-positive but the coverage was too low to be considered as negative; FN: false negative; TN: true negative; FP: false positive; TP: true positive). Please note that the current negative-defining methods do not affect positive mutation statuses (TP and FP).

Figures

Figure S1. Two-measurement simulation to evaluate the performance of negative-defining methods.

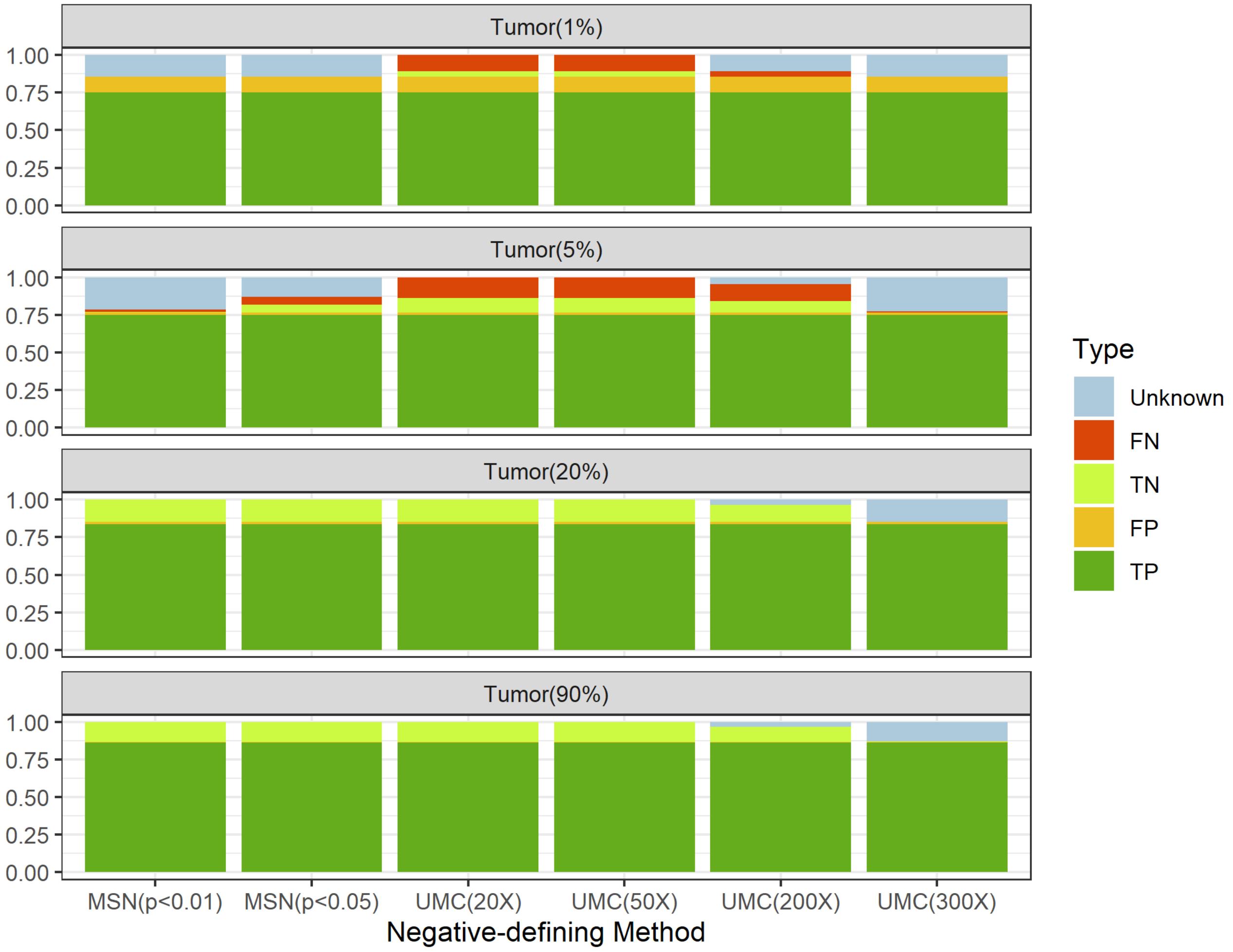

Supplement: Supplementary file 2 — Additional file 2. Supplementary figures. Figure S1: Two-measurement simulation to evaluate the performance of negative-defining methods. [file 12920_2021_880_MOESM2_ESM.pdf]
